# Comparison of amitriptyline supplemented with pregabalin, pregabalin supplemented with amitriptyline, and duloxetine supplemented with pregabalin for the treatment of diabetic peripheral neuropathic pain (OPTION-DM): a multicentre, double-blind, randomised crossover trial

**DOI:** 10.1016/S0140-6736(22)01472-6

**Published:** 2022-08-27

**Authors:** Solomon Tesfaye, Gordon Sloan, Jennifer Petrie, David White, Mike Bradburn, Stephen Julious, Satyan Rajbhandari, Sanjeev Sharma, Gerry Rayman, Ravikanth Gouni, Uazman Alam, Cindy Cooper, Amanda Loban, Katie Sutherland, Rachel Glover, Simon Waterhouse, Emily Turton, Michelle Horspool, Rajiv Gandhi, Deirdre Maguire, Edward B Jude, Syed H Ahmed, Prashanth Vas, Christian Hariman, Claire McDougall, Marion Devers, Vasileios Tsatlidis, Martin Johnson, Andrew S C Rice, Didier Bouhassira, David L Bennett, Dinesh Selvarajah, OPTION-DM trial group

**Affiliations:** a Diabetes Research Unit, Sheffield Teaching Hospitals NHS Foundation Trust, Sheffield, UK; b Clinical Trials Research Unit, University of Sheffield, Sheffield, UK; c Medical Statistics Group, University of Sheffield, Sheffield, UK; d School of Health and Related Research, and Department of Oncology and Metabolism, University of Sheffield, Sheffield, UK; e Department of Diabetes, Lancashire Teaching Hospitals NHS Trust, Chorley, UK; f Diabetes and Endocrine Centre, East Suffolk and North Essex NHS Foundation Trust, Ipswich, UK; g Diabetes and Endocrine Centre, Nottingham University Hospitals NHS Trust, Nottingham, UK; h Department of Cardiovascular & Metabolic Medicine, Institute of Life Course and Medical Sciences, University of Liverpool, Liverpool, UK; i School of Medicine, University of Liverpool, Liverpool, UK; j Liverpool University Hospital NHS Foundation Trust, Liverpool, UK; k NHS Sheffield Clinical Commissioning Group, Sheffield, UK; l Department of Diabetes and Endocrinology, Harrogate and District NHS Foundation Trust, Harrogate, UK; m Department of Diabetes and Endocrinology, Tameside and Glossop Integrated Care NHS Foundation Trust, Ashton under Lyne, UK; n Division of Diabetes, Endocrinology & Gastroenterology, University of Manchester, Manchester, UK; o Department of Diabetes and Endocrinology, Countess of Chester Hospital NHS Foundation Trust, Chester, UK; p Department of Diabetes, King's College Hospital NHS Foundation Trust, London, UK; q Department of Diabetes and Endocrinology, The Royal Wolverhampton NHS Trust, Wolverhampton, UK; r Department of Medicine, University Hospital Hairmyres, NHS Lanarkshire, Hairmyres, UK; s Department of Diabetes, University Hospital Monklands, NHS Lanarkshire, Monklands, UK; t Department of Endocrinology and Diabetes, Gateshead Health NHS Foundation Trust, Gateshead, UK; u St Pancras Clinical Research, London, UK; v Pain Research, Department of Surgery and Cancer, Faculty of Medicine, Imperial College London, London, UK; w Inserm U987, APHP, UVSQ, Paris-Saclay University, Paris, France; x Nuffield Department of Clinical Neurosciences, University of Oxford, Oxford, UK

## Abstract

**Background:**

Diabetic peripheral neuropathic pain (DPNP) is common and often distressing. Most guidelines recommend amitriptyline, duloxetine, pregabalin, or gabapentin as initial analgesic treatment for DPNP, but there is little comparative evidence on which one is best or whether they should be combined. We aimed to assess the efficacy and tolerability of different combinations of first-line drugs for treatment of DPNP.

**Methods:**

OPTION-DM was a multicentre, randomised, double-blind, crossover trial in patients with DPNP with mean daily pain numerical rating scale (NRS) of 4 or higher (scale is 0–10) from 13 UK centres. Participants were randomly assigned (1:1:1:1:1:1), with a predetermined randomisation schedule stratified by site using permuted blocks of size six or 12, to receive one of six ordered sequences of the three treatment pathways: amitriptyline supplemented with pregabalin (A-P), pregabalin supplemented with amitriptyline (P-A), and duloxetine supplemented with pregabalin (D-P), each pathway lasting 16 weeks. Monotherapy was given for 6 weeks and was supplemented with the combination medication if there was suboptimal pain relief (NRS >3), reflecting current clinical practice. Both treatments were titrated towards maximum tolerated dose (75 mg per day for amitriptyline, 120 mg per day for duloxetine, and 600 mg per day for pregabalin). The primary outcome was the difference in 7-day average daily pain during the final week of each pathway. This trial is registered with ISRCTN, ISRCTN17545443.

**Findings:**

Between Nov 14, 2017, and July 29, 2019, 252 patients were screened, 140 patients were randomly assigned, and 130 started a treatment pathway (with 84 completing at least two pathways) and were analysed for the primary outcome. The 7-day average NRS scores at week 16 decreased from a mean 6·6 (SD 1·5) at baseline to 3·3 (1·8) at week 16 in all three pathways. The mean difference was –0·1 (98·3% CI –0·5 to 0·3) for D-P versus A-P, –0·1 (–0·5 to 0·3) for P-A versus A-P, and 0·0 (–0·4 to 0·4) for P-A versus D-P, and thus not significant. Mean NRS reduction in patients on combination therapy was greater than in those who remained on monotherapy (1·0 [SD 1·3] *vs* 0·2 [1·5]). Adverse events were predictable for the monotherapies: we observed a significant increase in dizziness in the P-A pathway, nausea in the D-P pathway, and dry mouth in the A-P pathway.

**Interpretation:**

To our knowledge, this was the largest and longest ever, head-to-head, crossover neuropathic pain trial. We showed that all three treatment pathways and monotherapies had similar analgesic efficacy. Combination treatment was well tolerated and led to improved pain relief in patients with suboptimal pain control with a monotherapy.

**Funding:**

National Institute for Health Research (NIHR) Health Technology Assessment programme.

## Introduction

Diabetic peripheral neuropathy affects about 50% of people with diabetes over their lifetime and approximately half of these present with neuropathic pain.^1^ Diabetic peripheral neuropathic pain (DPNP) is associated with symptoms of burning, electric-shock type, lancinating, and deep-aching pains in the feet and legs and later in the upper limbs.^1^ Moderate-to-severe unremitting pain is present in over 70% of patients with DPNP resulting in insomnia, poor quality of life, mood disorders,^2^ and 5-times increased health-care costs compared with diabetes alone.^1^, ^3^ Chronic hyperglycaemia and cardiovascular risk factors have been shown to increase the risk of diabetic peripheral neuropathy,^2^ but the risk factors for DPNP and its pathophysiology are not fully understood.^1^

**Research in context**

**Evidence before this study**

The American Academy of Neurology (AAN) published a practice guideline on oral and topical treatments of diabetic peripheral neuropathic pain (DPNP). The panel searched the MEDLINE, Cochrane, Embase, and ClinicalTrials.gov databases from Jan 1, 2008, to April 30, 2020, for relevant peer-reviewed randomised controlled trials applying a careful analytical process to identify only class 1 and 2 studies. On July 12, 2022, we searched PubMed and Google Scholar with the key words “painful diabetic neuropathy” and identified two comparator randomised controlled trials published since April, 2020. Both trials were underpowered and used subtherapeutic doses of first-line drugs. The AAN report identified gaps in current knowledge including the following: few studies alone have investigated the effect of interventions on quality of life, patient functioning, mood, or sleep; there are few comparator studies of first-line drugs and their combinations; and scarce data is available on which patients respond to a specific intervention. The additional studies we identified did not address these limitations.

**Added value of this study**

Our large, multi-period crossover study compared the efficacy of three of the most prescribed first-line drugs for DPNP and their combinations not only for the primary outcome of pain relief, but also for important secondary outcomes including quality of life, mood, and sleep. We showed that all three treatment pathways and monotherapies had similar analgesic efficacy.

**Implications of all the available evidence**

To our knowledge, this was the largest and longest ever, head-to-head, multi-period crossover neuropathic pain trial. Our study can have an impact on future treatment guidelines, not only for DPNP, but also for chronic neuropathic pain treatment in general (estimated to affect 8–9% of the UK population) because current treatment guidelines are largely generic (eg, National Institute for Clinical Excellence's CG173). Our study can also benefit general practitioners, diabetes specialists, pain specialists, neurologists, general physicians, other medical and allied health-care professionals managing DPNP, and patients by the provision of improved treatment pathways. Some of the first-line drugs might not be available in resource-limited countries due to cost and other reasons, but our study can give confidence that any of these drugs or drug combinations, if titrated carefully to maximum tolerated doses, can result in similar levels of pain relief.

Most international guidelines recommend amitriptyline, duloxetine, pregabalin, or gabapentin as first-line agents for symptomatic analgesic therapy in patients with DPNP. Robust evidence exists for the efficacy of each drug based on Cochrane reviews and meta-analyses, but the best outcome for any monotherapy is 50% pain relief in fewer than half of patients, which is often accompanied by dose-limiting side-effects.^4^ The management of DPNP is hampered by the absence of robust, head-to-head evidence regarding which first-line agent to use first and which alternative agent to add in combination, when pain relief on monotherapy is suboptimal.^5^ The COMBO-DN study showed that the standard dose combination treatment of duloxetine and pregabalin had equivalent efficacy to maximum dose monotherapy of either drug.^6^ Moreover, combinations of tricyclic antidepressants and a gabapentinoid were found to be more efficacious than monotherapy.^7^, ^8^ However, these studies were small and had short treatment periods.^7^, ^8^ As a result, most current guidelines do not recommend combination treatment due to insufficient evidence^4^, ^5^ despite widespread use by clinicians. The absence of evidence-based treatment pathways results in increased patient suffering and health-care costs.^3^ Therefore, this context presents a good rationale for seeking robust evidence from well designed, head-to-head comparator trials of treatment pathways (first-line drugs and their combinations). Hence, the aim of the OPTION-DM trial was to determine the most clinically beneficial and best tolerated treatment pathway for patients with DPNP.

## Methods

### Study design

OPTION-DM was a multicentre, randomised, double-blind, centre-stratified, multi-period crossover trial with active washout in patients with DPNP from primary and secondary care at 13 UK centres. The Yorkshire and the Humber Sheffield Research Ethics Committee (16/YH/0459) approved the trial, an independent Trial Steering Committee oversaw the trial, and a Data Monitoring and Ethics Committee monitored safety. The study protocol is available online.

### Participants

Eligible participants were aged 18 years or older and fulfilled the diagnostic criteria for diabetes according to WHO^9^ (in the presence of diabetes symptoms, a random plasma glucose ≥11·1 mmol/L, fasting plasma glucose ≥7·0 mmol/L, or a 2 h plasma glucose ≥11·1 mmol/L with an oral glucose tolerance test; with no symptoms, another of these tests is required), had distal symmetrical polyneuropathy^10^ confirmed by the modified Toronto Clinical Neuropathy Score (score ≥5),^11^ and had daily neuropathic pain confirmed by the Douleur Neuropathique 4 questionnaire^12^ (score ≥4) for at least 3 months. All participants provided written informed consent.

Inclusion criteria were the following: average daily pain intensity of at least 4 over 7 days on the numerical rating scale (NRS; 0 indicating “no pain” and 10 indicating “worst pain imaginable”) while off pain medication, aspartate aminotransferase and alanine aminotransferase concentrations lower than twice the upper limit of normal, an estimated glomerular filtration rate (eGFR) of 30 mL/min per 1·73 m^2^ or higher, and stable glucose control over the preceding 3 months with glycated haemoglobin concentrations of 12% (108 mmol/mol) or lower. Included patients had sufficient cognitive and language skills to be able to comply with all the study requirements and be available for the duration of this year-long study.

Exclusion criteria included the following: a history of epilepsy, depression requiring antidepressant medications, pregnancy and breastfeeding, severe systemic disease, postural hypotension (systolic blood pressure drop >20 mm Hg on standing for 3 min), cardiac arrhythmias and conduction abnormalities on 12-lead electrocardiogram at baseline (as per usual clinical practice, electrocardiogram was not done during or after each treatment pathway), prostatic hypertrophy based on clinical history (eg, urinary frequency and urgency, trouble starting a urine stream, weak stream and dribbling at the end of urination, or urinary retention) and review of medical records, other painful peripheral neuropathies (B12, folate, and thyroid stimulating hormone checked if not documented in medical records over the preceding 12 months), the concomitant presence of other painful medical conditions that were as severe as their DPNP, major amputations of the lower limbs, active diabetic foot ulcers, and substantial suicide risk.^13^ All participants did not use analgesic medications and antidepressants, other than trial drugs and rescue medication paracetamol up to 1 g every 6 h if required, for the duration of the study.

### Randomisation and masking

The OPTION-DM trial examined three 16-week treatment pathways: oral amitriptyline supplemented with pregabalin (A-P), pregabalin supplemented with amitriptyline (P-A), and duloxetine supplemented with pregabalin (D-P; appendix p 9). Allocation concealment was achieved with site staff using the Sheffield Clinical Trials Research Unit's online randomisation system, which registered participant details before revealing allocation. The trial statistician created a predetermined randomisation schedule stratified by site using permuted blocks of size six or 12. Patients were randomly assigned 1:1:1:1:1:1 to receive one of six sequences over 50 weeks, comprising the three treatment pathways: A-P followed by D-P followed by P-A, A-P followed by P-A followed by D-P, D-P followed by A-P followed by P-A, D-P followed by P-A followed by A-P, P-A followed by D-P followed by A-P, and P-A followed by A-P followed by D-P. Masking of trial medication was maintained with identical over-encapsulated drugs and matching double-dummy placebo. The treating physician was masked to the treatment pathway but not the dose level. Those assessing outcomes and analysing data were also masked to the treatment pathways.

### Procedures

Each pathway had two treatment phases, each with a 2-week initial titration period (appendix p 9). During the first treatment phase, participants received monotherapy for 6 weeks. At the week 6 follow-up visit, on the basis of a 7-day average daily pain NRS score, those achieving mild pain (ie, NRS <3) were classed as responders, and those with NRS score higher than 3 were classed as non-responders.^14^ Responders continued monotherapy alone, reflecting clinical practice, whereas non-responders started the second drug in combination for an additional 10 weeks. Monotherapy and combination treatment doses were escalated towards the maximum tolerated dose or the target NRS of 3 or lower for satisfactory pain relief, whichever was reached first (appendix p 9). If first-line treatment was stopped due to an adverse reaction, participants were switched to the second-line agent until the end of the pathway. This study design is based on current clinical practice to ensure study outcomes can be readily applicable.

After the screening visit, analgesic medications were washed out over a period of 1–2 weeks, after which baseline daily NRS pain was recorded in paper diaries for 1 week. Participants with an average NRS score of 4 or higher were eligible for randomisation.^14^ A 1-week washout period between treatment pathways was implemented, as in previous studies (appendix p 9).^7^, ^8^ Trial medications were prescribed and titrated with three dose levels for each drug (appendix p 9). Amitriptyline was titrated to a maximum of 75 mg per day,^4^ duloxetine to 120 mg per day,^4^, ^6^ and pregabalin to 600 mg per day if the eGFR was 60 mL/min per 1·73 m^2^ or higher.^4^, ^6^ Patients with an eGFR of 30–59 mL/min per 1·73 m^2^ received a pregabalin dosing schedule starting with 75 mg per day, then 150 mg per day, and with a final dose of 300 mg per day.^15^ Details and schedule of the assessments required during the course of the treatment pathways are presented in the appendix (pp 10–11). This schedule was repeated from week 0 to week 16 for each pathway, until all three pathways were complete.

### Study outcomes

Participants recorded daily pain in paper diaries for the duration of the trial. The primary outcome was the difference in 7-day average NRS daily pain measured during the final follow-up week (week 16) between the treatment pathways.^16^ The difference in 7-day average daily NRS pain scores at week 6 between monotherapies was a secondary endpoint. Other secondary efficacy endpoints were quality of life measured by the RAND 36-item short-form survey (SF-36),^17^ the Hospital Anxiety and Depression Scale (HADS),^18^ the proportion of patients achieving 30% and 50% pain reduction from baseline, Brief Pain Inventory-Modified Short Form (BPI-MSF)^19^ items, the Insomnia Severity Index (ISI) total score,^20^ the Neuropathic Pain Symptom Inventory (NPSI) questionnaire total score and its five subscores,^21^ tolerability based on a 0–10 NRS scale, the Patient's Global Impression of Change (PGIC)^22^ at week 16, and patients’ preferred treatment pathway at trial conclusion at week 50. The onset, severity, and duration of adverse events were recorded in patient diaries. This was collected at each study visit and evaluated for severity and causality in relation to study medications. A cost-effectiveness analysis was also undertaken and will be reported elsewhere.

### Statistical analysis

The trial was initially powered on a mean difference between treatment pathways of 0·5 points on the NRS scale, on the basis of the effect size previously reported for comparison of two active interventions in a four-arm neuropathic pain crossover trial.^23^ Assuming a within-patient SD of 1·65, α=0·0167 to allow for three comparisons, 90% power, and 25% dropout, 392 participants would be required. However, recruitment and retention for this year-long demanding trial, with multiple study visits and four washout periods became challenging (appendix p 9), especially during the COVID-19 pandemic, and difficult to justify given that most previous similar trials^7^, ^8^, ^24^ had used a 1 NRS point difference, which is half of a clinically important amount of pain reduction.^25^ With the approval of the Trial Steering Committee, our Patient and Public Involvement panel, and the funder, a decision was made to continue the trial until an adequate sample size was achieved to detect a difference of at least 1 NRS point between treatment pathways.^7^ Using the original assumptions, 74 evaluable participants would provide 90% power to detect a difference of 1 NRS point and were sufficient to estimate differences in average pain to within an SE of 0·25 NRS points.

For the statistical analysis, we used a modified intention-to-treat approach, which included all eligible participants who started each treatment pathway. We analysed the primary outcome and other continuous outcomes using a linear mixed model, with treatment group, period, and the interaction between the two as fixed effects and participant as a normally distributed random intercept. If the interaction (ie, the carrying over of the treatment to the next treatment period) effect was not significant, then it was removed, and a reduced model was fitted. Residuals were plotted against treatment, pathway, and fitted values, whereas random effects were visualised in a dotplot. We used linear contrasts to evaluate differences between treatment groups. We compared the percentage of participants reporting adverse events using a global χ^2^ test across treatment groups, derived from a mixed effects logistic regression, with covariates being treatment group and period. Preplanned subgroup analyses according to baseline characteristics (age, sex, anxiety or depression, self-reported pain, and COVID-19 restrictions) were done descriptively by either Forest or Lowess plots and quantitatively by adding an interaction term in the statistical model; additional pre-planned analyses examined the change from baseline in pain among patients starting combination therapy compared with those who remained on monotherapy. Week-16 pain was the average pain between 106 and 112 days after the start of the treatment pathway or as close to this as possible within a window of –3 to +1 weeks, providing that this did not extend into the washout period and that scores were available for at least 4 of 7 consecutive days. Week-6 pain was scored analogously within a window of –2 to +1 weeks. Additionally, we assessed the effect of missing outcome data for the primary endpoint using multiple imputation, controlled multiple imputation,^26^ and last observation carried forward (LOCF). Multiple imputation incorporated NRS data from all 16 weeks and used predictive mean matching with ten nearest neighbours with age, sex, baseline NPSI, treatment, and period as covariates (appendix p 1). Controlled imputation used the values of delta between 0 (equivalent to missing at random) to +2·5 NRS units for participants who discontinued treatment due to toxicity or poor response. We assessed convergence using trace plots. As three pairwise comparisons were done, all statistical tests were two-tailed at 1·67% significance level, and 98·33% CIs were used for the difference between treatment pathways. Analyses were done with Stata, version 16.1. This trial was registered with ISRCTN, ISRCTN17545443, and EUDRACT, 2016–003146–89.

### Role of the funding source

The funders of the study had no role in study design, data collection, data analysis, data interpretation, or writing of the report.

## Results

**Figure 1:**
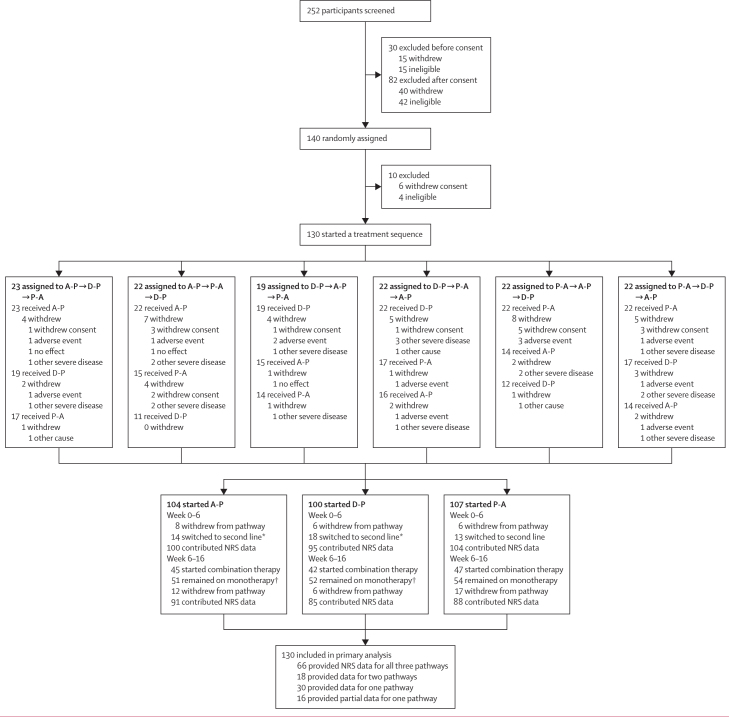
Trial profile A-P=amitriptyline supplemented with pregabalin. D-P=duloxetine supplemented with pregabalin. NRS=numerical rating scale. P-A=pregabalin supplemented with amitriptyline. *One participant in A-P and two participants in D-P switched and then withdrew before week 6. †One participant switched from first-line to second-line monotherapy.

Participants were recruited between Nov 14, 2017, and July 29, 2019. Follow-up continued until July 24, 2020. 252 patients were screened, with 140 randomly assigned to six treatment sequences, of whom 130 were included in the analysis (figure 1). 130 patients started a first pathway, 97 started a second pathway, and 84 started a third pathway. Mean age was 61·8 years (SD 11·0), similar to previous DPNP trials.^6^, ^7^ Previous neuropathic medication use was similar for amitriptyline, pregabalin, and duloxetine, and trial completers and non-completers had similar demographic characteristics (table 1). Additionally, we observed no differences in these variables between each of the six randomisation sequences (appendix p 2).

**Table 1:** Baseline demographic, diabetes, previous medication uses, and pain intensity characteristics of participants

		**Completers (n=77)**	**Non-completers (n=53)**	**Total (n=130)**
**Demographics**				
Age, years		61·0 (55–69)	61·0 (57–72)	61·0 (55–70)
Sex		..	..	..
	Female	22 (29%)	12 (23%)	34 (26%)
	Male	55 (71%)	41 (77%)	96 (74%)
Ethnicity		..	..	..
	White	72 (94%)	50 (94%)	122 (94%)
	Asian	3 (4%)	2 (4%)	5 (4%)
	Black	1 (1%)	1 (2%)	2 (2%)
	Mixed	1 (1%)	0	1 (1%)
BMI, kg/m^2^		31·7 (6·3)	31·7 (7·0)	31·7 (6·6)
**Diabetes characteristics**				
Type 1 diabetes		12 (16%)	10 (19%)	22 (17%)
	Missing	2 (3%)	0	2 (2%)
HbA_1c_, mmol/mol		65·4 (13·2)	68·4 (17·2)	66·6 (15·0)
Duration of diabetes, years		14·9 (9·0)	15·6 (9·7)	15·1 (9·3)
Duration of neuropathic pain, years		4·8 (4·1)	5·0 (4·1)	4·9 (4·1)
**Previous medication use**				
Amitriptyline		30 (39%)	19 (36%)	49 (38%)
Pregabalin		27 (35%)	18 (34%)	45 (35%)
Duloxetine		28 (36%)	19 (36%)	47 (36%)
Gabapentin		27 (35%)	17 (32%)	44 (34%)
Any opioids		27 (35%)	20 (38%)	47 (36%)
**Pain intensity**				
NRS pain*		6·7 (1·5)	6·5 (1·4)	6·6 (1·5)

Data are mean (SD), median (IQR), or n (%). HbA_1c_=glycated haemoglobin. NRS=numerical rating scale.

* Scale of 0–10; higher scores indicate greater pain.

**Figure 2:**
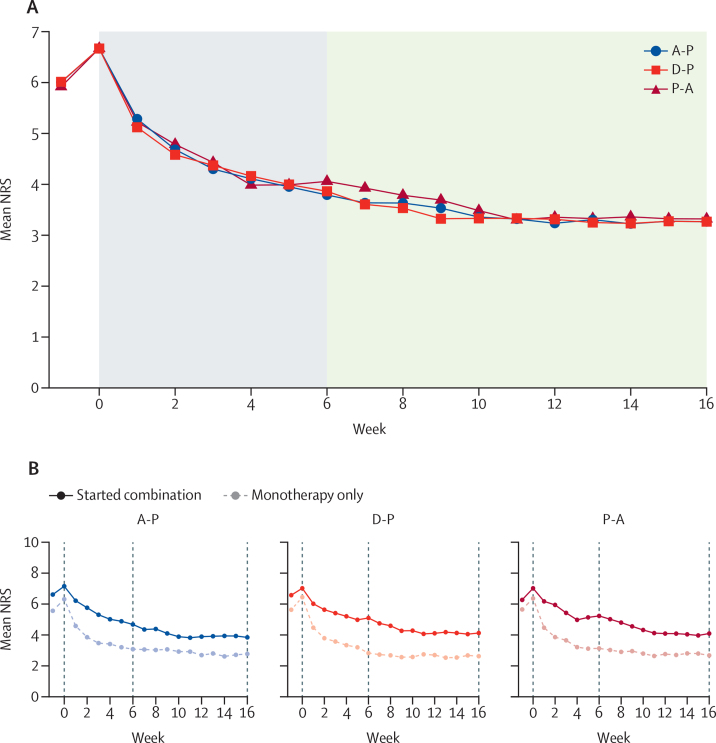
Mean daily pain intensity of the treatment pathways (A) and mean daily pain intensity in each treatment pathway comparing participants who started combination therapy with those remaining on monotherapy (B) Each treatment pathway contained a 6-week monotherapy phase and 10-week combination treatment phase for participants with NRS higher than 3. The introduction of study medications began with a 2-week titration period to achieve maximum tolerated dose. There was a 7-day washout period between treatment pathways. A-P=amitriptyline supplemented with pregabalin. D-P=duloxetine supplemented with pregabalin. NRS=numerical rating scale. P-A=pregabalin supplemented with amitriptyline.

We observed improvements in the 7-day average daily NRS pain at week 16 for all three treatment pathways, with no significant differences between them (primary endpoint; table 2). Among participants who completed their pain diary entries, NRS scores decreased from a mean 6·6 ( SD 1·5) at baseline to 3·3 (1·8) at week 16 in all three pathways, with slightly higher values observed when imputing with LOCF and controlled multiple imputation: the mean difference was –0·1 (98·3% CI –0·5 to 0·3) for D-P versus A-P, –0·1 (–0·5 to 0·3) for P-A versus A-P, and 0·0 (–0·4 to 0·4) for P-A versus D-P (table 2). These findings were robust across a range of analyses assessing missing data under the plausible scenario in which the 47 (15%) instances of missing data were imputed either by LOCF, multiple imputation, or controlled multiple imputation; additionally, we observed no significant main effects of treatment sequence or period and no evidence of carryover (p=0·90). Pain scores were also similar at week 6 and throughout all treatment pathways (figure 2A).

**Table 2:** Response to treatment by maximum tolerated doses of monotherapies at 6 weeks and at the end of the treatment pathways at 16 weeks, by intention-to-treat analysis

	**Baseline (n=130)**	**Week 6 monotherapy**					**Week 16 combination therapy**				
		A (n=104)	D (n=100)	P (n=107)	Mean difference (98·3% CI)	p value	A-P (n=104)	D-P (n=100)	P-A (n=107)	Mean difference (98·3% CI)	p value
**Average weekly pain**											
Participants	130	100	95	104	..	..	91	85	88	..	..
NRS pain*	6·6 (1·5)	3·8 (2·0)	3·9 (1·9)	4·1 (2·1)	..	..	3·3 (1·8)	3·3 (1·8)	3·3 (1·8)	..	..
Change from baseline	..	2·9 (2·0)	2·8 (2·0)	2·5 (2·2)	..	..	3·4 (2·1)	3·5 (2·1)	3·3 (2·1)	..	..
>30% reduction†	..	68 (65%)	63 (63%)	60 (56%)	..	..	68 (65%)	68 (68%)	68 (64%)	..	..
>50% reduction†	..	42 (40%)	35 (35%)	43 (40%)	..	..	50 (48%)	46 (46%)	47 (44%)	..	..
NRS <3†‡	..	38 (37%)	32 (32%)	36 (34%)	..	..	50 (48%)	43 (43%)	50 (47%)	..	..
**Pairwise contrast**											
D-P *vs* A-P	..	..	..	..	0·1 (−0·3 to 0·5)	0·65	..	..	..	−0·1 (−0·5 to 0·3)	0·61
P-A *vs* A-P	..	..	..	..	0·3 (−0·1 to 0·8)	0·049	..	..	..	−0·1 (−0·5 to 0·3)	0·61
P-A *vs* D-P	..	..	..	..	0·3 (−0·2 to 0·7)	0·14	..	..	..	0·0 (−0·4 to 0·4)	1·00

Data are n, mean (SD), or n (%); pairwise comparisons are mean difference (98·3% CI). NRS pain is rated on a scale of 0–10, with increasing numbers indicating increasing pain. A=amitriptyline. A-P=amitriptyline supplemented with pregabalin. D=duloxetine. D-P=duloxetine supplemented with pregabalin. NRS=numerical rating scale. P=pregabalin. P-A=pregabalin supplemented with amitriptyline.

* Measured for 7 days at baseline and for 7 days at maximum tolerated dose at weeks 6 and 16.

† Percentages assume participants with missing data were non-responders.

‡ NRS <3 is equivalent to mild pain reported by responders.

The mean maximum tolerated doses per day and the number (percentage) of participants on the maximum dose at week 6 were 56 mg (53 [51%] participants) for amitriptyline, 76 mg (46 [46%]) for duloxetine, and 397 mg (59 [55%]) for pregabalin. For patients on combination treatments, the mean maximum tolerated doses per day and the number (percentage) of participants on maximum dose of the second-line drug at week 16 were 347 mg (21 [47%] participants) for A-P, 405 mg (23 [55%]) for D-P, and 52 mg (22 [47%]) for P-A.Averaged across all treatment pathways, mean reduction in pain was 2·6 (98·3% CI 2·2 to 3·0) at week 6 (ie, monotherapy effect; n=299; p<0·0001) and 3·4 (2·9 to 3·8) at week 16 (n=265; p<0·0001). Patients who started combination therapy (ie, had inadequate response to monotherapy) saw a further reduction of 1·0 (SD 1·3) points (98·3% CI 0·6 to 1·3, p<0·0001) between weeks 6 and 16, whereas those who remained on monotherapy saw a mean pain reduction of 0·2 (1·5) points (98·3% CI –0·1 to 0·5, p=0·085; figure 2B, appendix p 12).

In total, 106 (35%) patients with outcome data responded to maximum tolerated monotherapy with an NRS of 3 or lower (ie, mild pain) and 120 (40%) achieved 50% reduction from baseline pain. Over the subsequent 10 weeks, combination treatment resulted in an additional 37 (19%) patients reaching an NRS of 3 or lower and 23 (14%) patients reaching 50% pain relief.

We assessed the results of study questionnaires (SF-36, HADS, and ISI) for participants receiving maximum tolerated doses of monotherapies at week 6 and at the end of the treatment pathways at week 16. All treatment pathways showed similar improvement from baseline in the SF-36 domains, HADS, ISI, and BPI-MSF items (table 3, appendix pp 3–8).

**Table 3:** SF-36, HADS, and Insomnia Severity Index at baseline, at week 6 and week 16

	**Baseline (n=130)**	**Monotherapy (week 6)**			**Combination therapy (week 16)**		
		A (n=93)	D (n=87)	P (n=99)	A-P (n=86)	D-P (n=86)	P-A (n=86)
**RAND SF-36 components**							
General health	38·2 (20·9)	38·4 (21·6)	37·2 (22·2)	39·8 (21·8)	35·2 (21·3)	36·9 (21·5)	36·9 (22·1)
Emotional wellbeing	63·1 (21·5)	66·5 (22·4)	68·7 (20·8)	66·7 (20·6)	67·6 (22·8)	66·5 (21·0)	66·3 (23·4)
Energy or fatigue	36·9 (20·4)	41·2 (21·8)	39·8 (22·7)	40·7 (21·8)	40·6 (22·7)	39·6 (21·4)	41·4 (21·3)
Pain	33·4 (20·5)	47·8 (21·9)	45·0 (20·6)	45·7 (23·2)	45·8 (25·5)	47·0 (24·5)	49·3 (23·8)
Physical functioning score	34·9 (26·9)	43·9 (27·4)	39·1 (28·3)	41·0 (28·1)	40·7 (26·9)	40·9 (30·1)	39·8 (28·6)
Role limitations due to emotional problems	41·3 (43·3)	50·5 (44·7)	51·7 (46·2)	54·2 (44·6)	46·9 (47·2)	48·1 (46·2)	53·5 (46·4)
Role limitations due to physical health*	21·9 (35·5)	28·8 (38·7)	23·0 (37·0)	30·3 (39·8)	26·5 (38·2)	25·9 (38·8)	26·7 (36·7)
Social functioning	49·3 (27·6)	60·8 (30·8)	58·0 (28·2)	61·1 (30·1)	57·8 (31·1)	58·3 (27·9)	60·3 (28·1)
Health change	..	65·6 (20·2)	65·8 (21·6)	59·1 (23·5)	67·4 (23·3)	61·0 (25·0)	62·8 (20·9)
Physical health component	20·5 (11·6)	25·4 (12·5)	22·6 (12·6)	24·5 (13·3)	23·6 (13·0)	24·1 (13·8)	24·1 (13·1)
Mental health component	44·9 (11·9)	46·7 (13·0)	47·8 (11·8)	47·6 (12·2)	46·6 (12·8)	46·3 (11·0)	47·4 (12·3)
**Mood and sleep**							
HADS–anxiety	8·7 (4·8)	7·5 (5·1)	7·4 (4·6)	6·7 (4·4)	7·7 (5·4)	7·3 (4·8)	7·0 (4·6)
HADS–depression	8·4 (4·6)	7·4 (4·7)	7·3 (4·4)	7·0 (4·5)	7·3 (4·9)	7·5 (4·5)	7·2 (4·5)
Insomnia Severity Index†	18·1 (5·9)	11·8 (7·3)	13·8 (6·3)	12·1 (7·1)	11·4 (7·3)	13·3 (6·8)	12·1 (6·4)

Data are mean (SD). A=amitriptyline. A-P=amitriptyline supplemented with pregabalin. D=duloxetine. D-P=duloxetine supplemented with pregabalin. HADS=Hospital Anxiety and Depression Scale. P=pregabalin. P-A=pregabalin supplemented with amitriptyline. SF-36=36-item short-form general health survey.

* Mean difference P-A *vs* D-P at week 6 of 7·6 (98·3% CI 0·4–14·9), p=0·011, and mean difference A-P *vs* D-P at week 6 of 7·3 (0·0–14·7), p=0·017.

† Mean difference D-P *vs* A-P at week 6 of 1·5 (0·0–3·1), p=0·016, and mean difference D-P *vs* A-P at week 16 of 1·5 (0·1–3·0), p=0·010.

Overall, we observed few pairwise differences in any of the questionnaire outcomes. The D-P pathway had lower scores for role limitation due to physical health than both P-A and A-P at week 6 but not at week 16, and higher insomnia (measured by the ISI score) than A-P at both week 6 and 16. However, these should be treated with caution given the number of secondary outcomes assessed. The PGIC questionnaire, completed at the end of each treatment pathway, showed that similar proportions of participants reported feeling “much improved” or “very much improved” (44% for A-P, 43% for D-P, and 49% for P-A, p=0·70). At the end of the study (week 50), the most preferred pathway was P-A (43%), followed by D-P (33%) and A-P (24%; p=0·27).

Assessing the planned subgroup analyses, we observed that patients with higher emotional distress at baseline showed greater improvement in pain scores with P-A and D-P compared with A-P (appendix p 13). Using the NPSI questionnaire, we also examined if different clinical pain phenotypes were associated with a better treatment response.^27^ 58 patients were defined as having deep pain, 45 having pinpointed pain, and 24 having evoked pain (three patients had incomplete baseline NPSI results and were excluded from the analysis). We observed no significant differences, and all mean NRS pain scores for each treatment pathway stratified by NPSI defined pain phenotypes were similar at week 6 and week 16 (appendix pp 4–6).

Table 4 shows treatment-emergent adverse events (TEAEs) reported by more than 5% of patients during monotherapy, combination therapy, and the treatment pathway as a whole. TEAEs were well recognised for the widely used study medications (dizziness was more common in the P-A pathway [p=0·036], nausea in the D-P pathway [p=0·0011], and dry mouth in the A-P pathway [p=0·0003]). We observed no significant differences in the reporting of serious adverse events between the treatment pathways. Overall, maximum tolerated dose combination treatment was generally well tolerated, with few discontinuations of either drug due to TEAEs (three [7%] of 45 with A-P, four [10%] of 42 with D-P, and five [11%] of 47 with P-A; p=0·88; figure 1). Most TEAE discontinuations occurred during the monotherapy phase, during which P-A had the fewest discontinuations (five [5%] of 107) compared with A-P (11 [11%] of 104) and D-P (17 [17%] of 100; p=0·031).

**Table 4:** Treatment-emergent adverse events reported in over 5% of patients during monotherapy (weeks 0–6, first 42 days), while on combination therapy (weeks 7–16, after 42 days), and on treatment pathway as a whole (weeks 0–16)

	**Monotherapy (weeks 0–6)**				**Combination therapy (weeks 7–16)**				**Treatment pathway (weeks 0–16)**			
	Amitriptyline (n=104)	Duloxetine (n=100)	Pregabalin (n=107)	p value	A-P (n=45)	D-P (n=42)	P-A (n=47)	p value	A-P (n=104)	D-P (n=100)	P-A (n=107)	p value
Fatigue	18 (17%)	17 (17%)	11 (10%)	0·25	4 (9%)	3 (7%)	9 (19%)	0·20	21 (20%)	18 (18%)	22 (21%)	0·88
Dry mouth	22 (21%)	5 (5%)	10 (9%)	0·036	10 (22%)	3 (7%)	9 (19%)	0·16	33 (32%)	8 (8%)	18 (17%)	0·0003
Dizziness	8 (8%)	8 (8%)	19 (18%)	0·029	5 (11%)	5 (12%)	4 (9%)	0·90	12 (12%)	16 (16%)	26 (24%)	0·036
Sedation	19 (18%)	6 (6%)	10 (9%)	0·021	2 (4%)	3 (7%)	5 (11%)	0·52	21 (20%)	11 (11%)	15 (14%)	0·17
Diarrhoea	8 (8%)	10 (10%)	6 (6%)	0·45	7 (16%)	6 (14%)	1 (2%)	0·16	18 (17%)	16 (16%)	9 (8%)	0·12
Nausea	4 (4%)	19 (19%)	6 (6%)	0·0042	1 (2%)	3 (7%)	2 (4%)	0·64	5 (5%)	23 (23%)	7 (7%)	0·0011
Oedema	2 (2%)	5 (5%)	14 (13%)	0·010	4 (9%)	3 (7%)	1 (2%)	NC	9 (9%)	10 (10%)	17 (16%)	0·15
Constipation	9 (9%)	8 (8%)	5 (5%)	0·57	3 (7%)	5 (12%)	2 (4%)	0·56	11 (11%)	13 (13%)	8 (7%)	0·47
Headaches	8 (8%)	10 (10%)	7 (7%)	0·68	1 (2%)	3 (7%)	0	NC	9 (9%)	14 (14%)	8 (7%)	0·33
Fall	3 (3%)	6 (6%)	5 (5%)	0·25	2 (4%)	4 (10%)	5 (11%)	0·20	7 (7%)	12 (12%)	10 (9%)	0·88
Excessive sweating	7 (7%)	7 (7%)	1 (1%)	0·14	1 (2%)	1 (2%)	5 (11%)	0·16	9 (9%)	10 (10%)	6 (6%)	0·58
Vomiting	5 (5%)	9 (9%)	1 (1%)	0·079	1 (2%)	2 (5%)	5 (11%)	NC	7 (7%)	11 (11%)	8 (7%)	0·51
Insomnia	3 (3%)	7 (7%)	3 (3%)	0·31	3 (7%)	2 (5%)	3 (6%)	0·85	6 (6%)	8 (8%)	7 (7%)	0·90
Abdominal cramping	4 (4%)	4 (4%)	3 (3%)	0·78	1 (2%)	0	1 (2%)	NC	5 (5%)	6 (6%)	4 (4%)	0·58
Ataxia	1 (1%)	2 (2%)	7 (7%)	0·091	3 (7%)	0	1 (2%)	NC	4 (4%)	4 (4%)	8 (7%)	0·41
Inability to concentrate	4 (4%)	1 (1%)	6 (6%)	0·23	1 (2%)	0	0	NC	5 (5%)	1 (1%)	6 (6%)	0·24

Data are n (%). Patients could report treatment emergent adverse events during monotherapy or combination therapy or both. Some p values could not be calculated with a model with both treatment and period as covariates. p values are for a global test across treatment groups. A-P=amitriptyline supplemented with pregabalin. D-P=duloxetine supplemented with pregabalin. NC=not calculated. P-A=pregabalin supplemented with amitriptyline.

## Discussion

The OPTION-DM trial showed that all three treatment pathways (A-P, D-P, and P-A) provided similar and significant pain reduction of 3·3 in NRS, or half the baseline pain intensity score. We found no statistically or clinically significant differences between the treatment pathways. To our knowledge, this was the first randomised, double-blind, comparator trial of neuropathic pain treatment pathways. Although head-to-head trials of individual monotherapies and combination treatments from the start could be designed, we felt that data on treatment pathways as a whole was most efficient and applicable to current clinical practice. This is because most patients are started on a monotherapy and will require a second agent added in combination within a few months. Therefore, OPTION-DM is a pragmatic trial, mirroring current neuropathic pain management pathways, allowing the outcomes of this study to be readily generalisable.

To our knowledge, OPTION-DM is the longest blinded neuropathic pain trial to date,^14^ with each patient undergoing all three treatment pathways over 50 weeks. Unlike previous combination-treatment crossover trials,^14^ the durations of monotherapy and combination treatments were sufficiently long to assess the full treatment effects, even though this resulted in higher-than-expected dropouts (84 completed at least two pathways) mainly for personal and other non-treatment-related reasons (73%). Moreover, previous combination trials used fixed-dose titration regimens regardless of treatment response,^6^, ^8^ which does not reflect clinical practice, and resulted in higher relative dropout rates.^28^ Our trial used a flexible dosing regimen to achieve maximum tolerated doses, based on individual responses.^7^, ^8^, ^23^

Although other tricyclic antidepressants are available, we used amitriptyline as it is the most widely prescribed tricyclic worldwide and a first-line agent in most guidelines.^4^, ^5^ We did not use gabapentin as there was little rationale for studying two α-2-δ ligands, and because it is a thrice-daily drug, does not have linear pharmacokinetics (unlike pregabalin), and requires a long titration period of up to 2 months to avoid toxicity.^1^, ^4^ We did not examine the pathway of pregabalin supplemented with duloxetine (P-D) because of the COMBO-DN findings, in which no difference in pain reduction was found if pregabalin was added to duloxetine or vice versa.^6^ However, duloxetine was better than pregabalin as an initial treatment at moderate doses and is a once-daily preparation, and thus we opted to examine the D-P pathway. Moreover, adding the P-D pathway would have prolonged the trial by 4 months.^6^ Finally, as both amitriptyline and duloxetine are antidepressants, there was little rationale for combining both.

Another strength of OPTION-DM is that it is, to our knowledge, the largest neuropathic pain crossover trial reported to date. All previous DPNP multiperiod, crossover trials had smaller sample sizes,^7^, ^8^, ^23^, ^24^, ^28^, ^29^ while several other studies included neuropathic pain conditions other than DPNP.^7^, ^8^, ^23^, ^24^ Early monitoring of the fidelity of recruitment and retention revealed that recruitment for this year-long, demanding trial was challenging. This was further compounded by the onset of the COVID-19 pandemic. Rather than compromise the integrity of the trial, and with the approval of the independent Trial Steering Committee, the trial continued until an adequate sample size had been achieved to detect a difference of at least 1 NRS point.^7^, ^25^ Given the identical NRS pain reduction of each pathway, even a significantly larger sample size, more than originally planned, would probably not have altered the principal study outcomes or conclusions.

Our trial also explored several secondary endpoints. The head-to-head comparison of the maximum tolerated doses of amitriptyline, duloxetine, and pregabalin showed similar efficacies for all three monotherapies, at the end of 6 weeks, although there were significantly less discontinuations due to TEAEs with pregabalin. However, monotherapy resulted in significant pain relief only in just over a third of participants (ie, responders, who reached NRS <3), and 50% pain relief in about 40%. Many patients (ie, non-responders) required combination treatment that resulted in a mean 1 point additional improvement in NRS (figure 2B, appendix p 12) and an additional 18% of patients reaching NRS lower than 3 and 14% reaching 50% pain relief. A crossover trial done in 56 patients with neuropathic pain (40 with DPNP) treated to maximum tolerated doses of gabapentin, nortriptyline, and their combination over 1-month^7^ treatment periods found that combination treatment was more efficacious than either drug alone. Another painful polyneuropathy crossover trial (n=73) of 5-week treatment periods compared the combination of imipramine and pregabalin at moderate doses to either treatment on its own.^8^ The study found that combination treatment was more efficacious in relieving neuropathic pain than each drug on its own, but resulted in higher rates of side-effects.^8^ Despite these results, several international bodies do not recommend combination treatment for DPNP due to insufficient evidence.^4^, ^5^ Although OPTION-DM was not designed as a comparison of monotherapy versus combination treatment, the data make a compelling case for the recommendation of combination treatment of first-line drugs for patients with DPNP with suboptimal response to a monotherapy (figure 3). The P-A pathway had the fewest discontinuations due to TEAEs, and although these results are not definitive, they suggest that the P-A pathway might be the best choice as a first-line treatment for DPNP.

Despite large variations in the cost and availability of amitriptyline, duloxetine, and pregabalin across the world, it is reassuring that all three are similarly efficacious in relieving pain. This will have real power to inform future clinical guidelines for the management of DPNP as available guidelines provide conflicting recommendations.^9^, ^30^, ^31^ Additionally, all monotherapies resulted in improvement of SF-36 quality-of-life domains, sleep, and measures of mood from baseline. However, amitriptyline was significantly better than duloxetine in improving physical functioning and sleep, and pregabalin was better than duloxetine in improving role limitation due to physical health. TEAEs were predictable for the monotherapies, although we observed no significant differences in frequency between the groups, which might partly be due to the use of maximum tolerated doses. Additionally, no significant differences were observed in the frequency of reported serious adverse events. However, compared with some previous studies,^8^ combination treatment with maximum tolerated doses was well tolerated with few TEAEs compared with monotherapy.

In this trial, the absence of a placebo group might be considered a limitation. However, these drugs are in common use all over the world, currently approved by regulatory and advisory bodies such as the National Institute for Health and Care Excellence on the basis of a large body of evidence for their efficacy from randomised placebo-controlled trials,^1^ meta-analyses,^4^ and Cochrane reviews. Furthermore, the addition of a placebo group would have increased the duration of this already long and demanding trial and, after a consultation with our Patient and Public Involvement Panel, we felt it was not ethically justifiable. Indeed, another crossover, combination trial without a placebo group showed a similar magnitude reduction in NRS pain for both monotherapy and combination treatments.^7^ Therefore, although some of the pain reduction will be due to the placebo effect alone, the magnitude of pain reduction is much greater than that achieved by placebo groups.^4^, ^5^

Another limitation of our study is the relatively high attrition, with only 59% of patients providing primary outcome data for all three pathways and 64% completing at least two pathways. The main reason for this was the long study duration (51 weeks), and thus the considerable demand on patients’ time (eg, delaying annual time off and so on). Nevertheless, our sensitivity analysis for the effect of missing data (including for-cause missing) suggests this primarily affected the precision of the difference rather than causing bias. Additionally, although a longer washout period (eg, 2 weeks) between treatment pathways would have been desirable, we felt this was unethical in this long and demanding trial and could have led to harm and even greater study discontinuations. Finally, although the study was not statistically powered to detect any carryover effect, this is unlikely to have had an effect on the primary outcome at the end of a long treatment period of 16 weeks.

In conclusion, the OPTION-DM trial showed that all three treatment pathways delivered similar analgesic efficacy both in terms of statistical and clinical significance. The trial also showed that combination treatment, where needed, was well tolerated and led to significantly better pain relief. Taken together, this study has great potential to influence treatment guidelines for DPNP.

## Data sharing

Requests for deidentified patient-level data and the statistical code should be made to the corresponding author, stating the data fields required and purpose of the request (ideally with a protocol but, at a minimum, with a research plan). The data dictionary and statistical analysis plan can also be made available. Requests will be considered on a case-by-case basis and requestors will be asked to complete a data sharing agreement with the sponsor before data transfer. Study records will be stored for 25 years after the completion of the study before being destroyed.

## Declaration of interests

ST reports honoraria for educational meetings from Pfizer, Viatris, Wörwag Pharma, Novo Nordisk, Merck & Co, Eva Pharma, Hikma Pharmaceuticals, Abbott Laboratories, AstraZeneca, Nevro, Procter & Gamble Health, Astellas Pharma, and Berlin-Chemi; consulting fees from Bayer, NeuroPn Therapeutics, Wörwag Pharma, Angelini, Grünenthal, TRIGOcare International, Nevro Mitsubishi Tanabe Pharma Corporation, and Confo Therapeutics; and a research grant from Procter and Gamble Health paid to Sheffield Teaching Hospitals. UA reports honoraria for educational meetings from Eli Lilly, Napp Pharmaceuticals, Sanofi, and Boehringer Ingelheim. EBJ reports honoraria and research support from Novo Nordisk and Sanofi. SHA reports honoraria for educational meetings from Novo Nordisk, Eli Lilly, and Sanofi. PV reports honoraria from Merck and Sanofi. MJ reports honoraria for advisory boards and speaker fees from Grünenthal (2015 to present) and being a co-chairperson, since 2014, of the Chronic Pain Policy Coalition and a council (2012 to present) and ordinary member of the British Pain Society. DB reports grants and contracts from Novartis, Grunenthal, Bayer, and Air Liquide; and consulting fees from Bayer and Air Liquide. DLB has acted as a consultant on behalf of Oxford University Innovation for AditumBio, Amgen, Bristows, Latigo Biotherapeutics, GlaxoSmithKline, Ionis Pharmaceuticals, Eli Lilly, OliPass, Regeneron Pharmaceuticals, and Theranexus, over the past 2 years; has received research funding from Eli Lilly and AstraZeneca; has received an industrial partnership grant from the Biotechnology and Biological Sciences Research Council and AstraZeneca; and reports grants and contracts for several studies from the UK Research and Innovation, Medical Research Council (MRC), Action Medical Research for Children, MRC Research Grant, Wellcome Trust Senior Clinical Scientist Fellowship, Novo Nordisk Foundation, EU Horizon 2020, MRC Clinical Research Training Fellowship, and Wellcome Trust Strategic Award. DS reports membership of the advisory boards of Impeto Medical, PelliTec, and FeetMe. CC is a member of the NIHR Clinical Trial Unit (CTU) Support Funding Committee, NIHR CTU Standing Advisory Committee, NIHR Programme Grants for Applied Research Subcommittee, and Trial Steering Committees for other NIHR-funded trials. All other authors declare no competing interests.
