
JOURNAL INFORMATION
==============================
NLM Title Abbreviation: Lancet
Journal ID: Lancet
NLM Journal ID: 2985213R

Lancet (London, England)

ISSN: 0140-6736
EISSN: 1474-547X

ARTICLE INFORMATION
==============================
PMCID: PMC9458867
PMID: 36049494
DOI: 10.1016/S0140-6736(22)01661-0
Article ID: S0140-6736(22)01661-0
Article version: 1
Subjects: Corrections

Department of Error

Print publication date: 2022 Sep 10
Volume: 400
Issue: 10355
First page: 810
Last page: 810
Copyright: © 2022 The Author(s). Published by Elsevier Ltd.
Copyright year: 2022
Copyright holder: Elsevier Ltd
License: This is an open access article under the CC BY license (http://creativecommons.org/licenses/by/4.0/).
License URL: https://creativecommons.org/licenses/by/4.0/
Erratum for: Comparison of amitriptyline supplemented with pregabalin, pregabalin supplemented with amitriptyline, and duloxetine supplemented with pregabalin for the treatment of diabetic peripheral neuropathic pain (OPTION-DM): a multicentre, double-blind, randomised crossover trial 400 10353 27 8 2022 680 690 Lancet (London, England) 10.1016/S0140-6736(22)01472-6 PMC9418415 36007534

==============================
Tesfaye S, Sloan G, Petrie J, et al. Comparison of amitriptyline supplemented with pregabalin, pregabalin supplemented with amitriptyline, and duloxetine supplemented with pregabalin for the treatment of diabetic peripheral neuropathic pain (OPTION-DM): a multicentre, double-blind, randomised crossover trial. Lancet 2022; 400: 680–90—In this Article, the spelling of author Steven Julious’ name was incorrect. This correction has been made to the online version as of Aug 29, 2022.

