# Supplementary material for: Comparison of amitriptyline supplemented with pregabalin, pregabalin supplemented with amitriptyline, and duloxetine supplemented with pregabalin for the treatment of diabetic peripheral neuropathic pain (OPTION-DM): a multicentre, double-blind, randomised crossover trial
Source: Lancet. 2022 Aug 27;400(10353):680–90. doi: 10.1016/S0140-6736(22)01472-6 (PMC9418415; doi:10.1016/S0140-6736(22)01472-6)
Supplement: Supplementary appendix [file mmc1.pdf]

THE LANCET

Supplementary appendix

This appendix formed part of the original submission and has been peer reviewed. We post it as supplied by the authors.

Supplement to: Tesfaye S, Sloan G, Petrie J, et al. Comparison of amitriptyline supplemented with pregabalin, pregabalin supplemented with amitriptyline, and duloxetine supplemented with pregabalin for the treatment of diabetic peripheral neuropathic pain (OPTION-DM): a multicentre, double-blind, randomised crossover trial. *Lancet* 2022; published online Aug 22. https://doi.org/10.1016/S0140-6736(22)01472-6.

Supplementary Material

Details of the multiple imputation models used to address missing NRS pain scores

'Missing pain scores were imputed using predictive mean matching with 10 nearest-neighbour matches. Imputations were chained across all weekly pain scores (i.e. not just weeks 6 and 16) and also the total NPSI score at weeks 6 and 16 within each pathway. The fixed (baseline) covariates used were baseline age, sex, treatment arm and treatment period (1,2,3). Convergence was assessed using trace plots which suggested a burn in of 1000 iterations.

Whilst the above method is valid under an assumption of missing at random, it is possible that some data were missing for-cause (ie missing not at random). Controlled imputation was used to assess the robustness of the findings to potentially informative missing data. The reason for treatment discontinuation and study discontinuation was recorded, and participants that did so citing poor tolerability and/or poor efficacy were identified as being potentially "for cause", or informatively missing. In these cases, sensitivity analyses were used to uplift imputed data by amounts between +0.5 and +2.5 NRS points. For example, a controlled imputation with delta=+0.5 meant adding 0.5 units onto imputed NRS scores (capped at a maximum of 10 points) for participants that discontinued their medication for those reasons.

We did not specify a hierarchy among the approaches for missing data, or for different values of delta but rather assessed consistency of findings across alternative assumptions.'

	A-P; D-P; P-A	A-P; P-A; D-P	D-P; A-P; P-A	D-P; P-A; A-P	P-A; A-P; D-P	P-A; D-P; A-P
n	23	22	19	22	22	22
Age, years	61.7 (10.8)	60.0 (11.8)	62.2 (13.2)	60.2 (9.7)	63.6 (11.2)	63.2 (10.1)
Female, n(%)	10 (43%)	8 (36%)	5 (26%)	3 (14%)	2 (9%)	6 (27%)
BMI, Kg/m^2	32.0 (7.4)	32.6 (6.8)	31.1 (5.9)	29.5 (5.7)	32.3 (6.9)	32.4 (6.7)
Type 1 diabetes, n(%)	5 (22%)	3 (14%)	3 (16%)	4 (18%)	4 (18%)	3 (14%)
HbA1c, mmol/mol	66.7 (12.9)	68.3 (13.6)	68.3 (14.9)	62.9 (18.3)	70.7 (16.5)	63.3 (13.3)
Diabetes duration, years [median(IQR)]	13.0 (5.2,19.9)	12.1 (8.5,17.6)	18.0 (10.8,22.4)	16.7 (11.6,20.6)	11.8 (6.1,15.6)	16.6 (10.3,20.8)
Pain duration, years [median(IQR)]	2.5 (1.5,5.0)	3.7 (2.5,5.0)	5.0 (1.8,8.0)	3.9 (2.0,7.0)	4.7 (2.4,6.0)	3.0 (2.0,8.0)
**Previous medication use n(%)**						
Amitriptyline	6 (26%)	6 (27%)	9 (47%)	8 (36%)	11 (50%)	9 (41%)
Pregabalin	10 (43%)	9 (41%)	6 (32%)	6 (27%)	10 (45%)	4 (18%)
Duloxetine	6 (26%)	9 (41%)	5 (26%)	7 (32%)	10 (45%)	10 (45%)
Gabapentin	6 (26%)	4 (18%)	11 (58%)	8 (36%)	8 (36%)	7 (32%)
Any opioid	8 (35%)	9 (41%)	9 (47%)	7 (32%)	8 (36%)	6 (27%)
Baseline pain (NRS)	6.9 (1.3)	6.4 (1.6)	7.0 (1.7)	6.7 (1.2)	6.8 (1.3)	6.1 (1.7)
BPI Pain severity score	6.0 (1.3)	6.0 (2.1)	6.3 (1.6)	6.1 (1.4)	6.5 (1.5)	5.6 (2.2)
Pain interference score	6.5 (1.8)	5.9 (2.4)	6.0 (2.8)	5.6 (2.0)	6.6 (2.4)	5.1 (2.7)
HADS Anxiety	10.9 (4.9)	8.0 (4.0)	8.9 (5.8)	8.0 (4.3)	9.9 (5.0)	6.6 (4.4)
HADS Depression	9.8 (3.8)	8.6 (4.2)	9.1 (5.3)	7.9 (5.2)	8.9 (4.2)	6.3 (4.7)

Table S1: Demographics, previous neuropathic pain medication use, pain and questionnaire scores by Treatment Sequence. A-P, amitriptyline supplemented by pregabalin; D-P, duloxetine supplemented by pregabalin and P-A; pregabalin supplemented by amitriptyline; BMI, body mass index; IQR, Interquartile range; NRS, numeric rating score; BPI, Brief Pain Inventory questionnaire; HADS, Hospital Anxiety and Depression Scale. All results mean (standard deviation) unless otherwise stated.

Baseline	Median (IQR)		
Pain at its worst in the last 24 hours	7.0 (6.0,8.0)		
Pain at its least in the last 24 hours	5.0 (3.0,7.0)		
Average pain	6.0 (5.0,7.0)		
Pain you have right now	6.0 (5.0,7.0)		
Pain severity score	6.2 (5.0,7.0)		
Pain interference score	6.1 (4.1,7.7)		
	**A-P (N=104)**	**D-P (N=100)**	**P-A (N=107)**
**Week 6**			
**Pain severity score**			
n	92	87	99
Median (IQR)	3.8 (2.3,5.0)	3.8 (3.0,5.0)	4.0 (2.8,5.8)
Pairwise comparisons	Mean difference (98.3% CI)	P	
D-P versus A-P	0.2 (-0.3, 0.7)	0.279	
P-A versus A-P	0.5 (0.0, 1.0)	0.01	
P-A versus D-P	0.3 (-0.2, 0.8)	0.17	
**Interference score**			
n	93	87	99
Median (IQR)	3.9 (1.9,5.7)	4.6 (2.0,6.3)	4.1 (2.1,6.6)
Pairwise comparisons	Mean difference (98.3% CI)	P	
D-P versus A-P	0.2 (-0.3, 0.8)	0.29	
P-A versus A-P	0.4 (-0.1, 1.0)	0.07	
P-A versus D-P	0.2 (-0.4, 0.7)	0.45	
**Week 16**			
**Pain severity score**			
n	86	86	86
Median (IQR)	3.1 (2.3,5.0)	3.5 (2.5,5.3)	3.3 (2.0,4.8)
Pairwise comparisons	Mean difference (98.3% CI)	p	
D-P versus A-P	0.0 (-0.4, 0.5)	0.97	
P-A versus A-P	-0.2 (-0.7, 0.2)	0.23	
P-A versus D-P	-0.2 (-0.7, 0.2)	0.22	
**Interference score**			
n	86	86	86
Median (IQR)	4.1 (2.0,6.1)	3.9 (2.1,6.1)	3.6 (1.4,5.9)
Pairwise comparisons	Mean difference (98.3% CI)	p	
D-P versus A-P	-0.1 (-0.7, 0.4)	0.56	
P-A versus A-P	-0.3 (-0.9, 0.2)	0.15	
P-A versus D-P	-0.2 (-0.7, 0.3)	0.39	

Table S2: Brief Pain Inventory (BPI) questionnaire results at baseline and at Week 6 and 16. Results presented as mean (standard deviation) unless otherwise stated.

Baseline	Median (IQR)		
Superficial spontaneous pain	7.0 (4.0,8.0)		
Missing	0		
Deep spontaneous pain	5.0 (3.0,7.0)		
Missing	2		
Paroxysmal pain	5.5 (3.5,7.5)		
Missing	0		
Evoked pains	4.0 (2.7,6.2)		
Missing	2		
Paraesthesia/dysesthesia	7.0 (5.0,8.0)		
Missing	1		
Total score	53.0 (38.0,67.0)		
Missing	3		
**Week 6**	**A-P**	**D-P**	**P-A**
**Superficial spontaneous pain**			
n	93	87	99
Median (IQR)	3.0 (1.0,6.0)	3.0 (1.0,6.0)	4.0 (1.0,6.0)
Pairwise comparisons	MD (98.3% CI)	p	
D-P versus A-P	0.1 (-0.6, 0.8)	0.66	
P-A versus A-P	0.6 (-0.1, 1.3)	0.05	
P-A versus D-P	0.4 (-0.3, 1.1)	0.13	
**Deep spontaneous pain**			
n	93	87	99
Median (IQR)	2.5 (0.5,4.5)	3.0 (0.5,4.5)	3.0 (0.5,5.0)
Pairwise comparisons	MD (98.3% CI)	p	
D-P versus A-P	-0.2 (-0.7, 0.4)	0.51	
P-A versus A-P	0.2 (-0.4, 0.8)	0.36	
P-A versus D-P	0.4 (-0.2, 1.0)	0.12	
**Paroxysmal pain**			
n	93	87	98
Median (IQR)	3.5 (1.0,5.5)	3.0 (1.5,5.0)	3.3 (1.5,5.5)
Pairwise comparisons	MD (98.3% CI)	p	
D-P versus A-P	-0.3 (-0.9, 0.4)	0.34	
P-A versus A-P	0.1 (-0.5, 0.7)	0.78	
P-A versus D-P	0.3 (-0.3, 0.9)	0.21	
**Evoked pain**			
n	93	85	98
Median (IQR)	2.3 (0.7,4.3)	2.3 (1.0,4.3)	2.7 (0.7,4.3)
Pairwise comparisons	MD (98.3% CI)	p	
D-P versus A-P	-0.1 (-0.6, 0.3)	0.55	
P-A versus A-P	0.1 (-0.4, 0.5)	0.65	
P-A versus D-P	0.2 (-0.3, 0.7)	0.29	
**Paraesthesia/dysesthesia**			
n	93	85	99
Median (IQR)	5.0 (2.0,6.0)	4.0 (2.5,6.0)	4.0 (2.0,7.0)
Pairwise comparisons	MD (98.3% CI)	p	
D-P versus A-P	-0.0 (-0.7, 0.7)	0.93	
P-A versus A-P	0.1 (-0.6, 0.8)	0.70	
P-A versus D-P	0.1 (-0.5, 0.8)	0.63	
**Total score**			

n	93	85	97
Median (IQR)	32.0 (16.0,47.0)	31.0 (20.0,45.0)	32.0 (17.0,49.0)
Pairwise comparisons	MD (98.3% CI)	p	
D-P versus A-P	-1.2 (-5.2, 2.9)	0.49	
P-A versus A-P	1.4 (-2.6, 5.4)	0.40	
P-A versus D-P	2.6 (-1.4, 6.6)	0.12	
**Week 16**	**A-P**	**D-P**	**P-A**
**Superficial spontaneous pain**			
n	86	86	86
Median (IQR)	2.0 (0.0,6.0)	4.0 (1.0,6.0)	3.0 (1.0,6.0)
Pairwise comparisons	MD (98.3% CI)	p	
D-P versus A-P	0.5 (-0.3, 1.3)	0.16	
P-A versus A-P	0.3 (-0.5, 1.1)	0.39	
P-A versus D-P	-0.2 (-1.0, 0.6)	0.57	
**Deep spontaneous pain**			
n	86	86	86
Median (IQR)	2.5 (0.0,5.0)	3.5 (1.0,5.0)	2.3 (0.5,5.5)
Pairwise comparisons	MD (98.3% CI)	p	
D-P versus A-P	0.5 (-0.1, 1.1)	0.05	
P-A versus A-P	0.3 (-0.3, 0.9)	0.20	
P-A versus D-P	-0.2 (-0.8, 0.4)	0.47	
**Paroxysmal pain**			
n	86	85	85
Median (IQR)	3.0 (0.0,6.0)	3.5 (1.5,6.0)	3.0 (1.0,6.0)
Pairwise comparisons	MD (98.3% CI)	p	
D-P versus A-P	0.3 (-0.3, 0.9)	0.18	
P-A versus A-P	0.1 (-0.5, 0.7)	0.68	
P-A versus D-P	-0.2 (-0.8, 0.4)	0.34	
**Evoked pain**			
n	86	86	85
Median (IQR)	3.0 (0.7,5.0)	2.7 (0.3,5.0)	2.7 (0.7,5.0)
Pairwise comparisons	MD (98.3% CI)	p	
D-P versus A-P	-0.0 (-0.6, 0.6)	0.95	
P-A versus A-P	0.0 (-0.6, 0.6)	0.99	
P-A versus D-P	0.0 (-0.6, 0.6)	0.94	
**Paraesthesia/dysesthesia**			
n	85	86	84
Median (IQR)	4.0 (2.0,6.5)	4.0 (1.5,6.0)	4.0 (2.0,6.8)
Pairwise comparisons	MD (98.3% CI)	p	
D-P versus A-P	-0.2 (-1.0, 0.6)	0.56	
P-A versus A-P	0.3 (-0.5, 1.1)	0.41	
P-A versus D-P	0.5 (-0.3, 1.3)	0.15	
**Total score**			
n	85	85	83
Median (IQR)	27.0 (14.0,53.0)	33.0 (20.0,48.0)	28.0 (17.0,50.0)
Pairwise comparisons	MD (98.3% CI)	p	
D-P versus A-P	1.9 (-3.0, 6.7)	0.36	
P-A versus A-P	1.6 (-3.2, 6.4)	0.44	
P-A versus D-P	-0.3 (-5.1, 4.5)	0.88	

Table S3: Neuropathic Pain Symptom Inventory (NPSI) Items at baseline, Week-6 and Week-16. Higher scores indicate greater pain intensity. MD, mean difference; SD, standard deviation; IQR, interquartile range. Results presented as mean (standard deviation) unless otherwise stated

	Monotherapy (week 6)		Combination therapy (week 16)	
	**Mean difference**	**p value**	**Mean difference**	**p value**
**RAND SF-36 components**				
General health				
D-P versus A-P	–1·7 (–5·4 to 2·1)	0·288	1·3 (–2·4 to 5·1)	0·397
P-A versus A-P	–0·8 (–4·5 to 2·9)	0·598	0·1 (–3·6 to 3·8)	0·961
P-A versus D-P	0·9 (–2·8 to 4·6)	0·581	–1·3 (–5·0 to 2·5)	0·418
Emotional wellbeing				
D-P versus A-P	2·6 (–1·6 to 6·9)	0·136	–1·9 (–6·3 to 2·5)	0·304
P-A versus A-P	–0·3 (–4·4 to 3·9)	0·884	–2·5 (–6·9 to 1·8)	0·164
P-A versus D-P	–2·9 (–7·1 to 1·3)	0·097	–0·6 (–5·0 to 3·7)	0·723
Energy / fatigue				
D-P versus A-P	–0·9 (–5·2 to 3·3)	0·597	0·1 (–4·3 to 4·5)	0·965
P-A versus A-P	0·5 (–3·7 to 4·6)	0·785	–0·0 (–4·4 to 4·3)	0·989
P-A versus D-P	1·4 (–2·8 to 5·6)	0·419	–0·1 (–4·4 to 4·2)	0·953
Pain				
D-P versus A-P	–2·7 (–7·2 to 1·9)	0·163	1·6 (–4·0 to 7·3)	0·488
P-A versus A-P	–2·4 (–6·8 to 2·1)	0·199	2·6 (–3·0 to 8·2)	0·275
P-A versus D-P	0·3 (–4·2 to 4·7)	0·889	0·9 (–4·7 to 6·5)	0·695
Physical functioning score				
D-P versus A-P	–4·3 (–9·0 to 0·5)	0·031	–0·4 (–5·4 to 4·6)	0·831
P-A versus A-P	–2·8 (–7·5 to 1·8)	0·143	–2·5 (–7·4 to 2·4)	0·225
P-A versus D-P	1·4 (–3·2 to 6·1)	0·463	–2·1 (–7·0 to 2·9)	0·317
Role limitations due to emotional problems				
D-P versus A-P	0·9 (–10·5 to 12·3)	0·856	1·6 (–9·7 to 12·8)	0·738
P-A versus A-P	4·8 (–6·3 to 15·9)	0·303	8·9 (–2·3 to 20·0)	0·057
P-A versus D-P	3·9 (–7·3 to 15·1)	0·403	7·3 (–3·8 to 18·4)	0·117
Role limitations due to physical health				
D-P versus A-P	–7·3 (–14·7 to 0·0)	0·017	–1·3 (–9·7 to 7·2)	0·721
P-A versus A-P	0·3 (–6·9 to 7·5)	0·917	–0·4 (–8·7 to 8·0)	0·918
P-A versus D-P	7·6 (0·4 to 14·9)	0·011	0·9 (–7·5 to 9·3)	0·796
Social functioning				
D-P versus A-P	–2·5 (–8·0 to 3·0)	0·274	0·8 (–5·1 to 6·7)	0·752
P-A versus A-P	0·0 (–5·3 to 5·4)	0·985	1·7 (–4·1 to 7·5)	0·489
P-A versus D-P	2·5 (–2·8 to 7·9)	0·258	0·9 (–4·9 to 6·7)	0·708
Health change				
D-P versus A-P	1·9 (–5·0 to 8·8)	0·517	–5·1 (–12·5 to 2·4)	0·104
P-A versus A-P	–5·2 (–11·8 to 1·5)	0·064	–3·7 (–11·1 to 3·7)	0·234
P-A versus D-P	–7·0 (–13·8 to –0·3)	0·013	1·4 (–6·0 to 8·8)	0·658
Physical health component				
D-P versus A-P	–2·9 (–4·9 to –0·9)	0·0004	0·4 (–1·9 to 2·7)	0·711
P-A versus A-P	–1·4 (–3·4 to 0·5)	0·078	–0·4 (–2·6 to 1·9)	0·699
P-A versus D-P	1·5 (–0·5 to 3·4)	0·067	–0·7 (–3·0 to 1·5)	0·444
Mental health component				
D-P versus A-P	1·3 (–1·0 to 3·6)	0·182	–0·2 (–2·5 to 2·2)	0·855
P-A versus A-P	1·1 (–1·1 to 3·4)	0·229	0·8 (–1·5 to 3·1)	0·417
P-A versus D-P	–0·1 (–2·4 to 2·1)	0·875	1·0 (–1·4 to 3·3)	0·317
**Mood and sleep**				

HADS - anxiety				
D-P versus A-P	–0·1 (–0·9 to 0·7)	0·826	–0·3 (–1·2 to 0·6)	0·449
P-A versus A-P	–0·5 (–1·3 to 0·3)	0·138	–0·4 (–1·4 to 0·5)	0·253
P-A versus D-P	–0·4 (–1·2 to 0·4)	0·213	–0·1 (–1·1 to 0·8)	0·705
HADS - depression				
D-P versus A-P	–0·2 (–1·0 to 0·6)	0·489	–0·1 (–0·9 to 0·6)	0·698
P-A versus A-P	–0·4 (–1·1 to 0·4)	0·274	–0·0 (–0·8 to 0·7)	0·903
P-A versus D-P	–0·1 (–0·9 to 0·7)	0·705	0·1 (–0·6 to 0·8)	0·786
Insomnia Severity Index				
D-P versus A-P	1·5 (0·0 to 3·1)	0·016	1·5 (0·1 to 3·0)	0·010
P-A versus A-P	0·5 (–1·0 to 2·0)	0·456	1·0 (–0·4 to 2·4)	0·082
P-A versus D-P	–1·1 (–2·6 to 0·4)	0·089	–0·5 (–1·9 to 0·9)	0·385

Pairwise comparisons are mean difference (98·3% CI). A-amitriptyline supplemented by pregabalin. D-duloxetine supplemented by pregabalin. HADS=Hospital Anxiety and Depression Score. P-A=pregabalin supplemented by amitriptyline. SF-36=short-form 36-item general health survey.

Table S4: Pairwise comparisons of treatment pathways at week 6 and week 16

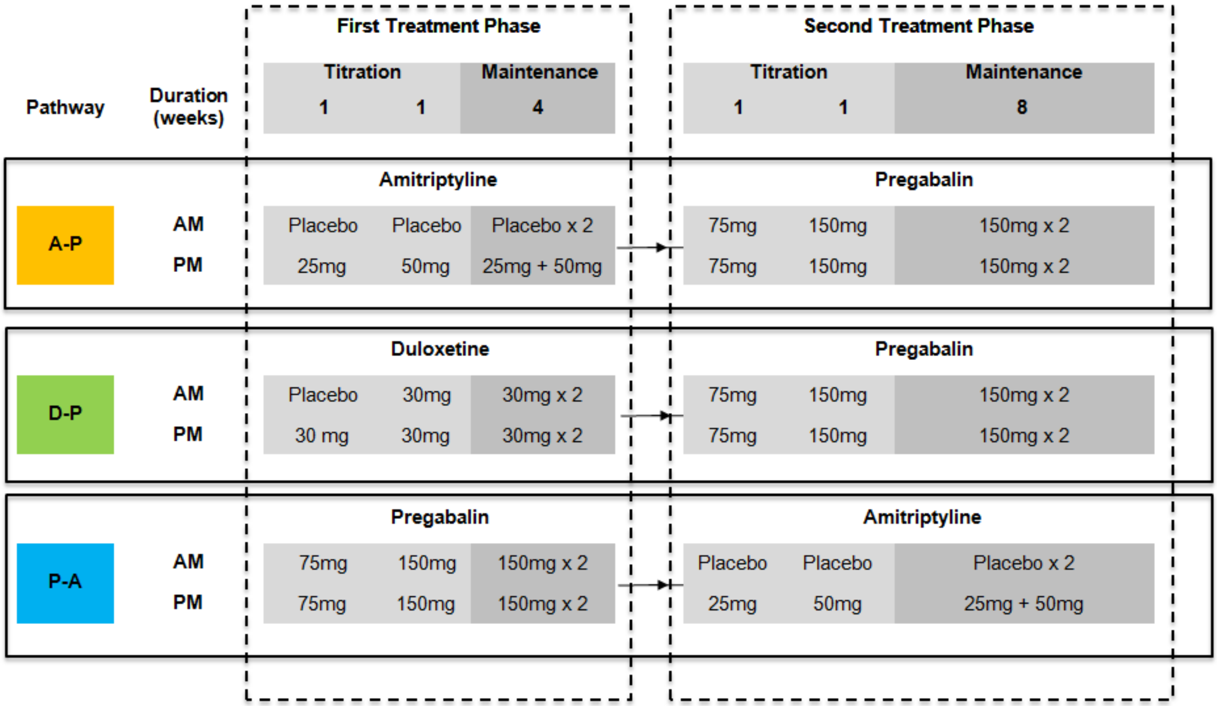

Figure S1: Dosing and titration schedule for Treatment Pathways: A-P (amitriptyline supplemented by pregabalin), D-P (duloxetine supplemented by pregabalin) and P-A (pregabalin supplemented by amitriptyline). Each pathway had two Treatment Phases, each with a 2-week initial titration period towards maximum tolerated dose. Participants continued on maximum tolerated maintenance dose of the drug from the first Treatment Phase for the duration of the second Treatment Phase. For patients with eGFR 30-59 ml/min/1.73m^2 the maximum pregabalin dose was 300mg/day.

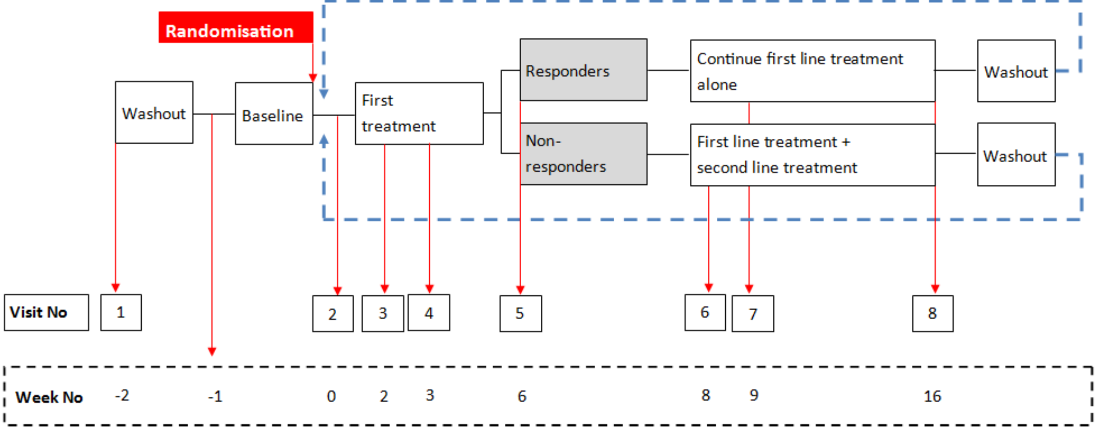

Figure S2: Patient flow chart through the first Treatment Pathway. Visits from week 0 to week 16 are repeated until all three pathways have been completed.

Assessments	Screening	Weeks from starting treatment pathway[b]							
	-2[a]	0[c]	2[c]	3[c]	6[c]	8[c,d]	9[c]	16[c, e]	17[f]
Informed consent	X								
Blood Tests[gh]	X							X	
ECG	X								
Medical History	X								
Physical and neurological assessment	X								
modified Toronto Clinical Neuropathy Score (mTCNS)	X								
Douleur Neuropathique 4 (DN4)	X								
Suicidal risk questionnaire	X								
Concomitant Medications	X	X	X	X	X	X	X	X	X
Vital Signs[i]	X							X	
Pregnancy Test (for women of child bearing potential)		X[k]		X	X		X	X	
Randomisation (treatment allocation)		X[k]							
Dispense Study Medication		X	X	X	X	X	X	X	
Pain Diaries[j]	X	X	X	X	X	X	X	X	
Tolerability scale		X[k]			X			X	
Brief Pain Inventory-Modified Short Form (BPI-MSF)		X[k]			X			X	
Insomnia Severity Index (ISI)		X[k]			X			X	
Neuropathy Pain Symptom Inventory (NPSI)		X[k]			X			X	
Hospital Anxiety and Depression Scale (HADS)		X[k]			X			X	
RAND Short Form 36 (RAND SF-36)		X[k]			X			X	
EQ-5D-5L		X[k]			X			X	
Client Service Receipt Inventory (CSRI)		X[k]			X			X	
Pain Catastrophising Scale (PCS)		X[k]							
Adverse Events Assessment		X[l]	X	X	X	X	X	X	X
Compliance Assessment		X[l]	X	X	X	X	X	X	X
Patient Global Impression of Change (PGIC)								X	

Figure S3: Study Assessment Schedule (SPIRIT Figure)

The study assessment schedule below details the assessments required during the course of one treatment pathway. All participants will complete 3 treatment pathways and this schedule will be repeated from week 0 to week 16 until all 3 pathways are complete. Week 17 will only be relevant at the end of the final pathway.

a. This visit is only required prior to randomisation i.e. before starting the first treatment pathway.
b. Between scheduled study visits, the research nurse will contact the participant by phone each week (a minimum of once per week). The nurse will confirm compliance with medication and remind the participant to complete study diaries/questionnaires.
c. Visits must normally be within +/- 2 days of the scheduled visit date. Scheduled visit dates relate to the date of the previous visit. Where this is impossible, e.g. due to Bank Holidays or patient availability.

d. Week 8 visit only required for participants on combination treatment.
e. At the week 16 visit, participants will be given instructions to taper off the current study treatment (see section 8.3.3 for details). Visits from week 0 to week 16 will be repeated until all 3 pathways have been completed.
f. Week 17 is only applicable following the final pathway.
g. FBC, urea and electrolytes, liver function tests, glycosylated haemoglobin A1c and serum creatinine.
h. Whole blood sample to be collected and stored for future research. The sample can be obtained at the same time as any scheduled blood test for the study. Please refer to the OPTION-DM Sample Collection Manual for details.
i. Height (at week -2 only), weight, heart rate and blood pressure (lying and standing).
j. To be completed by participants daily during the study, starting during the washout period. Pain scores may also be collected via daily text messages where participants have given additional consent for this.
k. Only required at week 0 of pathway 1 i.e. randomisation visit.
l. Not required at week 0 of pathway 1 i.e. randomisation visit.

A

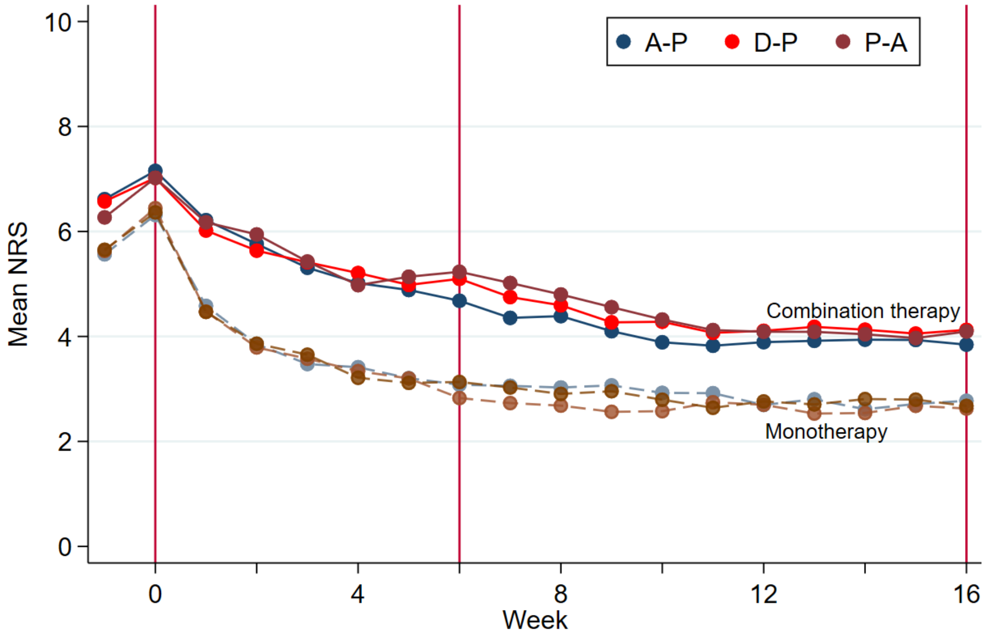

	A-P	D-P	P-A	All
Started combination therapy	0.9 (0.4, 1.4)	0.9 (0.4, 1.5)	1.1 (0.5, 1.6)	1.0 (0.6, 1.3)*
Remained on monotherapy	0.1 (-0.4, 0.6)	0.2 (-0.3, 0.7)	0.3 (-0.2, 0.8)	0.2 (-0.1, 0.5)*
Overall change by arm	0.5 (0.1, 0.8)	0.5 (0.2, 0.9)	0.7 (0.3, 1.0)	0.6 (0.3, 0.8)

B

**Combined arms**	**n=299**	**n=265**
Δ baseline	2.6 (2.2, 3.0); p<0.001	3.4 (2.9, 3.8); p<0.001*
≥50% reduction, n (%)	120 (40%)	143 (54%)
NRS ≤3, n (%)	106 (35%)	143 (54%)
Δ **Week-6 to 16**		
Combination therapy	1.0 (0.6, 1.3)**	
Monotherapy	0.2 (-0.1, 0.5)**	
All patients	0.6 (0.3, 0.8)	

Figure S4: A, Trajectory of pain response by Treatment Pathway showing significant change when combination treatment is initiated. A-P, amitriptyline supplemented by pregabalin, D-P, duloxetine supplemented by pregabalin and P-A, pregabalin supplemented by amitriptyline. **Table:** Mean (98.3% CI) change in pain numeric rating score (NRS) from week 6 to week 16 by treatment and use of combination therapy. * Patients that started combination therapy (i.e. had inadequate response to monotherapy) saw a further reduction of 1.0 (SD 1.3) points ((p<0.001; 98.3% CI 0.6:1.3) between weeks 6-16 whilst those that remained on monotherapy saw a mean pain reduction of 0.2 (1.5) points (p<0.001; 98.3% CI-0.1:0.5). **B** *p<0.001 for the difference

between the combined arms of monotherapy and combination treatment. **p<0.001 for the difference between Weeks 6 to 16 on combined monotherapy and combination treatment

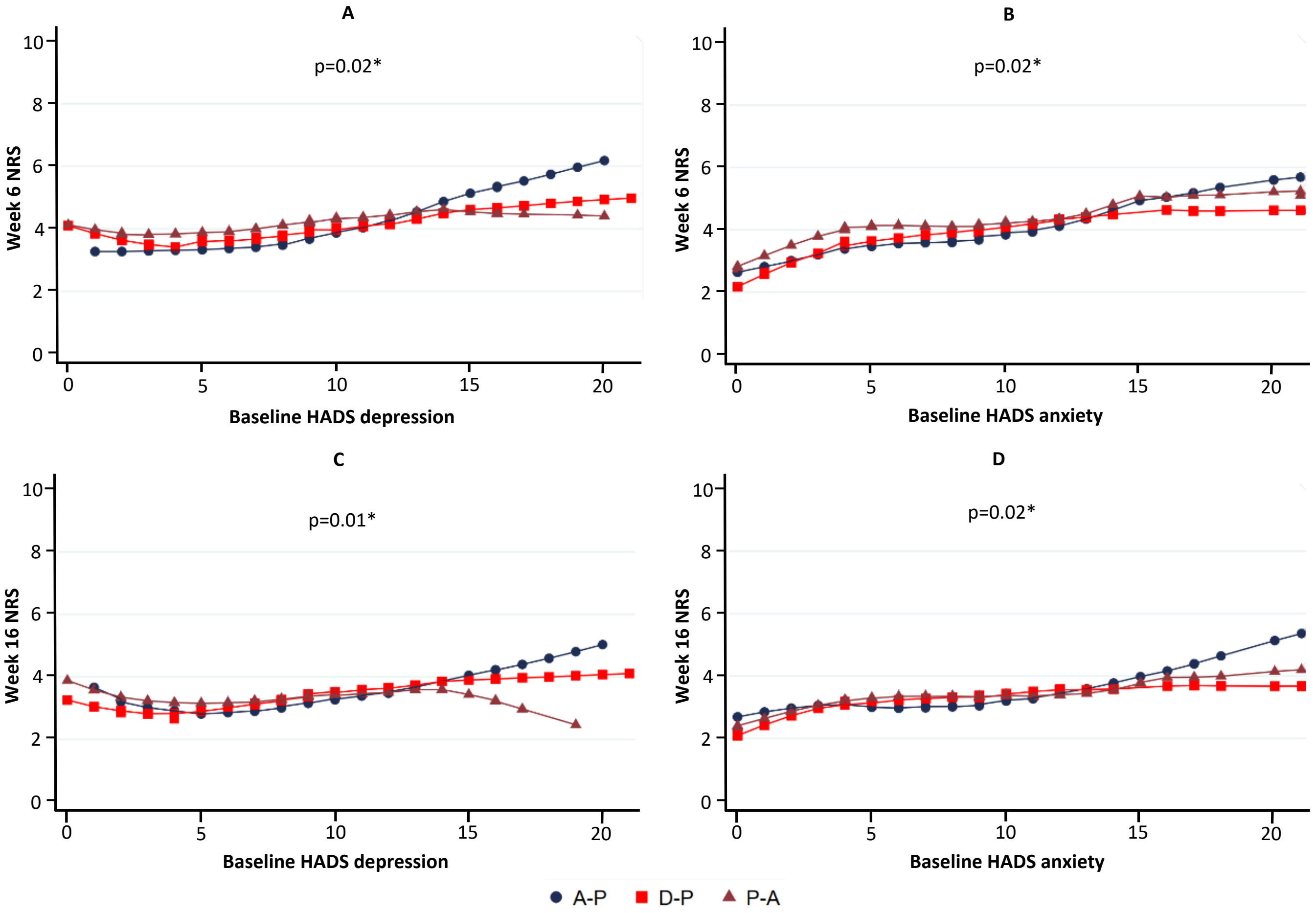

Figure S5: Lowess smoothed plot of numeric rating scale (NRS) pain scores at Week 6 according to baseline Hospital Anxiety and Depression Scale (HADS) scores for A, depression and B, anxiety and at Week 16 for C, depression and D, anxiety. Patients with higher baseline levels of emotional distress showed significant improvement in mean NRS-pain scores with the P-A and D-P Treatment Pathways compared to A-P. A-P, amitriptyline supplemented by pregabalin, D-P, duloxetine supplemented by pregabalin and P-A, pregabalin supplemented by amitriptyline. * p-value for interaction.
